# Phenotypic and functional testing of circulating regulatory T cells in advanced melanoma patients treated with neoadjuvant ipilimumab

**DOI:** 10.1186/s40425-016-0141-1

**Published:** 2016-06-21

**Authors:** Janet Retseck, Robert VanderWeele, Hui-Min Lin, Yan Lin, Lisa H. Butterfield, Ahmad A. Tarhini

**Affiliations:** University of Pittsburgh Cancer Institute, UPMC Cancer Pavilion, 5150 Centre Avenue (555), Pittsburgh, PA 15232 USA

**Keywords:** Melanoma, Ipilimumab, CTLA4, Regulatory T cells

## Abstract

**Background:**

We have previously investigated neoadjuvant ipilimumab (ipi) for patients with locally/regionally advanced melanoma. That initial assessment of peripheral blood mononuclear cells (PBMC) showed a significant increase in shared tumor associated antigen specific CD4^+^ and CD8^+^ T cell activation. We also observed a transient increase in circulating T regulatory cells (Treg) with a parallel increase in total CD4^+^ T cells, as well as a significant decrease in circulating myeloid derived suppressor cells (MDSC). The increase in circulating Treg frequency, as assessed at 6 weeks after initiation of ipilimumab, was significantly associated with improved progression free survival (PFS, *p* = 0.034; HR = 0.57) and returned to baseline levels by 12 weeks. To shed light on the unexpected positive correlation between increased Treg and PFS, we here investigated the suppressive activity of circulating Treg at baseline and 6 weeks.

**Methods:**

Patients were treated with ipi (10 mg/kg intravenously every 3 weeks for 2 doses) bracketing definitive surgery. Treg (CD4^+^CD25^+^CD127^dim/-^) were isolated from pre-ipi (baseline) and post-ipi (6 weeks) PBMC samples. Treg were co-cultured with autologous responder CD4^+^ T cells that were stimulated with OKT3/IL-2/CD28 and CFSE-labeled T cells. 1:1, 1:2, and 1:5 ratios were tested. Flow cytometery was used to evaluate the degree of Treg proliferation suppression.

**Results:**

Thirty-five patients were enrolled in the study; 18 patients had adequate PBMC samples with sufficient Treg isolated for Treg functional analysis. At 6 weeks following ipi, a decrease in percent of maximal inhibition of Th by Treg compared to baseline was seen for some patients. Scatter plot analysis showed no association between Treg frequency and function at any ratio or between circulating Treg frequency and function at baseline and at 6 weeks post-ipi. An increase in Treg suppressive function was significantly associated with a decrease in PFS (*p* = 0.02).

**Conclusions:**

We find that Treg frequency measures do not correlate with suppressive activity measured ex vivo. Treg suppressive activity increases correlate with poorer patient outcomes.

**Electronic supplementary material:**

The online version of this article (doi:10.1186/s40425-016-0141-1) contains supplementary material, which is available to authorized users.

## Background

Locally and regionally advanced melanoma has a high recurrence and mortality rate, with a 5-year survival for Stage IIIA, IIIB, and IIIC disease of 78 %, 59 %, and 40 %, respectively [1]. At the time of this study, the only Food and Drug Administration (FDA)-approved post-operative adjuvant treatment for high risk melanoma (Stages IIB - III) following wide local excision and lymph node dissection was interferon alpha-2b (IFN) or ipilimumab at 10 mg/kg. Approval of IFN was granted based on the results of four clinical trials. In E1684, IFN was dosed at 20 million units (MU)/m^2^/day intravenously for 1 month followed by 11 months of maintenance IFN at 10 MU/m^2^ SQ three times a week vs. observation alone. At median follow up of 6.9 years (for *n* = 280), both relapse-free survival (RFS) (HR 0.61; *p* = 0.0013) and overall survival (OS) (HR 0.67; *p* = 0.0115) were significantly better for IFN vs. observation [2]. High-dose interferon was subsequently shown to have superior RFS to low-dose interferon and to observation in E1690 [3] and to have superior RFS and OS to the GM2 ganglioside vaccine GMK in E1694 [4]. Pegylated IFN has also been shown to improve RFS as compared to placebo in the EORTC 18071 trial [5]. IFN remained the only FDA-approved adjuvant treatment for high-risk melanoma for many years. However, toxicities associated with IFN can lead to frequent dose reductions or significant shortening of the length of treatment [6]. The toxicity attrition rate ranged from 26 % in E1684 to 10 % in E1694.

Ipilimumab (ipi) is a monoclonal antibody directed against the immune checkpoint molecule CTLA-4, which was approved by the FDA in 2011 at the dose of 3 mg/kg for use in patients with advanced inoperable melanoma (inoperable Stages III and IV) [7]. Overall survival can be durable; a pooled analysis of 1861 patients in clinical trials showed a median survival of 11.4 months (95 % CI 10.7–12.1 months). There was a plateau in the survival curve at 3 years, with up to 10 years of follow up. The survival rate at 3 years was 22 % for both previously treated and treatment-naive patients who had received ipi [8]. More recently, adjuvant ipi at 10 mg/kg was shown to improve RFS of patients with stage III melanoma as compared to placebo in the EORTC trial 18071 (HR 0.75, 95 % CI 0.64 – 0.90) [9].

Tumors evade the immune response by many mechanisms, including the generation of an immune suppressive environment both systemically and at the tumor site. This is mediated in part by recruiting regulatory T cells (Treg), which can recognize tumor-associated antigens and expand, leading to suppression of anti-tumor effector and helper T cells (Th). These cells can suppress in an antigen-specific and non-specific manner and secrete suppressive soluble factors [10]. CTLA-4 is a negative regulator of T cell activation and proliferation [11]. In down-regulating the immune response, it keeps autoimmunity in check [12]. CTLA-4 expression is upregulated on activated T cells, whereas CTLA-4 is constitutively expressed on CD4^+^CD25^+^ Treg [13]. CTLA-4 expressed by CD4^+^CD25^hi^Foxp3^+^ Treg is thought to contribute to suppression of T effector cells (Teff) and Th, as CTLA-4 deficiency has been shown to decrease both self-tolerance and suppressive function of CD4^+^CD25^+^ Treg in tumor immunity [14]. It has been shown that CTLA-4 blockade of Teff increased Th function, while CTLA-4 blockade of Treg decreased Treg suppressive function, and that both are necessary for the anti-tumor activity of therapeutic CTLA-4 antibodies [15]. If Treg function can be suppressed through CTLA-4 blockade with an agent such as ipi, then an immune response to tumor antigens can potentially emerge and expand.

We have previously investigated neoadjuvant ipi for patients with locally and regionally advanced melanoma [16]. Patients underwent tumor biopsy prior to ipi and tumor resection after 2 doses of ipi at 10 mg/kg IV three weeks apart. Following ipi, there was a significant increase in CD8^+^ tumor infiltrating lymphocytes (TIL) as determined by immunohistochemistry (*n* = 24; *p* = 0.02). In a subset of patients, TIL immune monitoring by flow cytometry was also performed (*n* = 10). We observed increased tumor infiltration following ipi by activated (CD69^+^) CD3^+^/CD4^+^ T cells (*p* = 0.06) and CD3^+^/CD8^+^ T cells (*p* = 0.2) compared to baseline. There was also evidence of induction/potentiation of memory T cells expressing cytokine (CD3^+^/CD8^+^/CD45RO^+^/TNFα^+^; *p* = 0.03) but not naive (CD3^+^/ CD8^+^/CD45RO^−^/TNFα^+^; *p* = 0.44) T cells at 6 weeks. There was a trend towards an inverse association between the change in Treg in tumor and clinical benefit (complete response/partial response/stable disease versus progressive disease; *p* = 0.09). CD4^+^CD25^hi^Foxp3^+^ Treg infiltration was higher at week 6 (mean change = 1.5; SD = 1.46) in the progressive disease group, and lower in the clinical-benefit group (mean change = −0.64; SD = 1.83).

Immune monitoring of the circulation was also performed as part of the study. Assessment of peripheral blood mononuclear cells (PBMC) showed a significant increase in the percentage circulating Treg (CD4^+^CD25^hi^Foxp3^+^ and CD4^+^CD25^hi^CD39^+^). Unexpectedly, a significant increase in circulating Treg (CD4^+^CD25hi^+^Foxp3^+^) was associated with improved outcome, while conversely, we observed a significant decrease in circulating myeloid derived suppressor cells (MDSC), also associated with improved outcome. MDSC are known to inhibit T cell frequency and activation. As expected with CTLA-4 blockade, the increase in Treg paralleled an increase in the total CD4^+^ T cell population [17]. We speculated that Treg suppressive function might be changed by ipi therapy, and that this might be discordant with the circulating frequency. Therefore, in this study, we tested the suppressive activity of circulating Treg.

## Methods

### Patients

The characteristics of the total patient population were reported in the previously published study [16]. Characteristics of the 18 tested here are shown in Table 1. Eligible patients were 18 years or older and had clinically detectable locally and/or regionally advanced melanoma (cutaneous, mucosal or unknown primary).

**Table 1:** Patient demographics and baseline disease characteristics (*N* = 18 patients)

Variable	No. of patients (%)
Age, years; median (range)	53 (30–73)
Cutaneous primary	17 (94)
Unknown primary	1 (6)
Gender	
Female	12 (67)
Male	6 (33)
Performance status (ECOG)^a^	
0	11 (61)
1	7 (39)
Recurrent disease after prior surgery	15 (83)
Prior adjuvant HDI^b^	6 (33)
Presence of in-transit metastases	12 (67)
Estimated risk stage	
IIIB	2 (11)
IIIC	16 (89)
Tumor mutational status	
BRAF^V600^	9 (50)
NRAS^Q61^	5 (28)
Unknown	1 (5)

^a^ECOG: Eastern Cooperative Oncology Group; ^b^HDI: high dose interferon-α

### Study design

Following excisional biopsy, patients received induction ipi at 10 mg/kg IV on Day 0 and Day 21, and then underwent complete lymph node dissection. Patients received maintenance ipi at 10 mg/kg IV 2–4 weeks following lymphadenectomy, for a total of 2 doses 3 weeks apart. Blood was drawn into heparin (for PBMC) tubes or tubes without anticoagulant (serum) and processed by the Immunologic Monitoring Lab upon receipt at baseline and at 6 weeks. PBMC were isolated by Ficoll gradient and cryopreserved for batched testing according to standard operating procedures (SOPs).

### Treg isolation and testing

The Miltenyi Biotec Treg isolation (CD4^+^CD25^+^CD127^dim/-^) kit was used to purify Treg according to manufacturer’s instructions. CD4^+^CD25^−^ Th cells were similarly collected and used as responder cells. These cells were labeled with carboxyl fluorescent succinimidyl ester (CFSE), and 96-well plates were coated with OKT3 and incubated for 2 h. CFSE-labeled responder cells without Treg were added to the negative control wells and were used as background for “minimal” level of proliferation. The remaining CFSE-labeled responder cells were stimulated with CD28 and IL-2 and added to appropriate wells. Positive controls were OKT3/CD28/IL-2 stimulated Th without Treg addition and were set to 100 % or “maximal” level of proliferation. The Treg were then added to corresponding wells at the different ratios. The plates were incubated for 5 days in 37 °C, 5 % CO_2_. Following incubation, cells were stained with surface markers CD4 R-Phycoerythrin (PE) PE-Cy5 and CD25 PE/Cy7 fluorescent dyes for flow cytometry. Representative control flow cytometry plots are shown in Additional file 1: Figure S1. Healthy donor control proliferation inhibition assays are shown in Additional file 2: Table S1, while Additional file 3: Table S2 provides data on Treg purification by flow. To compare data between patients, each blood sample was normalized to 0 % baseline proliferation and 100 % proliferation without Treg (maximum), with each Treg ratio falling in between (Table 2).

**Table 2:**
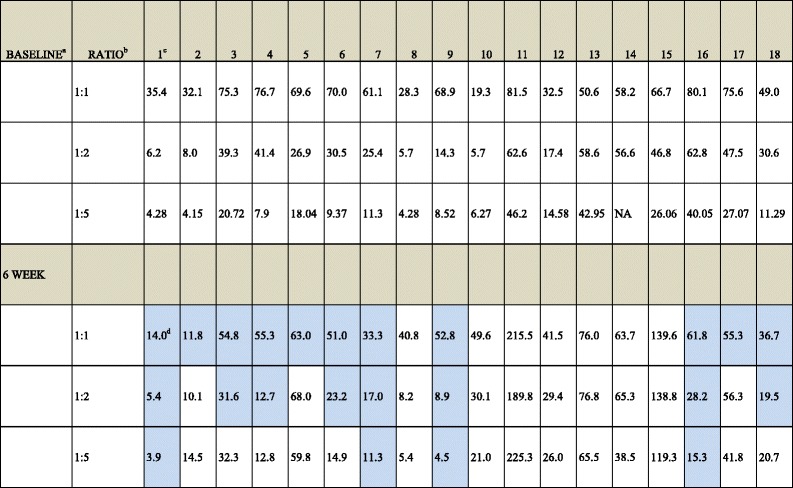
Percent proliferation inhibition by Treg at different Treg:Th ratios at either baseline or 6 week timepoints

^a^Timepoint; ^b^Treg:Th ratio; ^c^Patient number; ^d^Results highlighted in blue represent a decrease in percent of maximal inhibition post-ipi compared to corresponding baseline sample

Daily FC500-flow cytometer QC was run using Beckman-Coulter Flow-Check, Flow-Check675 and Flow-Check770 for laser alignment verification. Beckman-Coulter Flow-Set fluorospheres were used to standardize voltages to ensure consistency from day-to-day. Single-stained Beckman-Coulter Immuno-Trol control cells were used to establish compensation settings. Beckman-Coulter CXP Software version 2.1 and Beckman-Coulter Kaluza Software version 1.2 were used.

### Statistical analysis

Spearman correlation coefficients were used to evaluate the correlation between change of Treg function (6 weeks-baseline) and the Treg frequency previously measured, as well as correlation between circulating Treg frequency and function at baseline and 6 weeks. We determined the direction of Treg function change at 6 weeks by comparing to baseline by the majority vote of the three ratios. Kaplan-Meier method was used to evaluate progression free survival (PFS), and the exact log rank test was used to compare the PFS between the patients whose Treg function decreased at 6 week after the treatment and those whose Treg function increased at 6 weeks.

## Results

Thirty-five patients were enrolled in the study. We previously reported the circulating frequencies of Treg and other cells in this study [16]. Eighteen patients had adequate remaining PBMC samples with sufficient Treg cells for functional analysis. CD4^+^CD25^+^CD127^low^ cells were purified and tested for proliferation inhibition (suppressive) function against patient autologous CD4+ T cells, which were stimulated with anti-CD3, anti-CD28, and IL-2. The percent of proliferation inhibition across several titrated Treg:Th ratios is shown in Table 2. Patients varied in the suppressive activity of their Treg. The range of proliferation inhibition at 1:1 Treg:Th was 19–81 %. At six weeks’ post-ipi, a decrease in the percent of maximal inhibition of Th by Treg compared to baseline (at 2 or 3 of the 3 tested Treg ratios) was seen for 11 of 18 patients (Table 2). When ratios were statistically examined separately, the change in Treg suppression after treatment was not significant for the highest Treg ratios of 1:1 (*p* = 0.1439) or 1:2 (*p* = 0.782). It was significant only for the 1:5 ratio (*p* = 0.02557, Additional file 4: Figure S2).

**Fig. 1:**
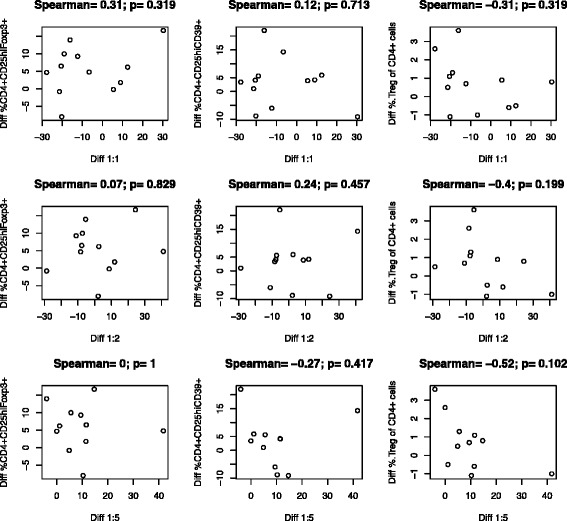
Association between the change in peripheral blood Treg frequency and Treg suppressive function (change in % Treg proliferation after the treatment) across different Treg:Th ratios. The change (week 6 level – baseline level) in circulating Treg was measured phenotypically as CD4^+^/CD25^hi^/Foxp3^+^ (Diff CD4^+^CD29^hi^Foxp3^+^, left column), CD4^+^/CD25^hi^/CD39 (Diff CD4^+^CD25^+^CD39^+^, middle column), or the percent of circulating Treg (Diff %Treg in CD4^+^ Cells, right column). The percent suppressive activity, calculated as the week 6 % Treg inhibition – baseline % Treg function, at 1:1 (Diff 1:1, top row), 1:2 (Diff 1:2, middle row), or 1:5 (Diff 1:5, bottom row) was plotted against the frequency of Treg across all patients with valid test results. There was no statistically significant association (p values on each part). Fewer than 18 data points were generated due to missing values on some patients

**Fig. 2:**
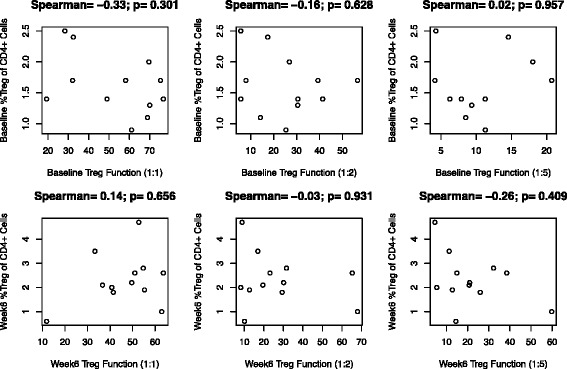
Association between circulating Treg frequency and Treg function (% Treg function, pre- and post-treatment). Baseline phenotypic (baseline circulating Treg cells) and suppressive function at each ratio (baseline %Treg function; top row), and post treatment measures (bottom row) were tested across patients for correlations. There was no significant association (p values on each part). Fewer than 18 data points were generated due to missing values on some patients

**Fig. 3:**
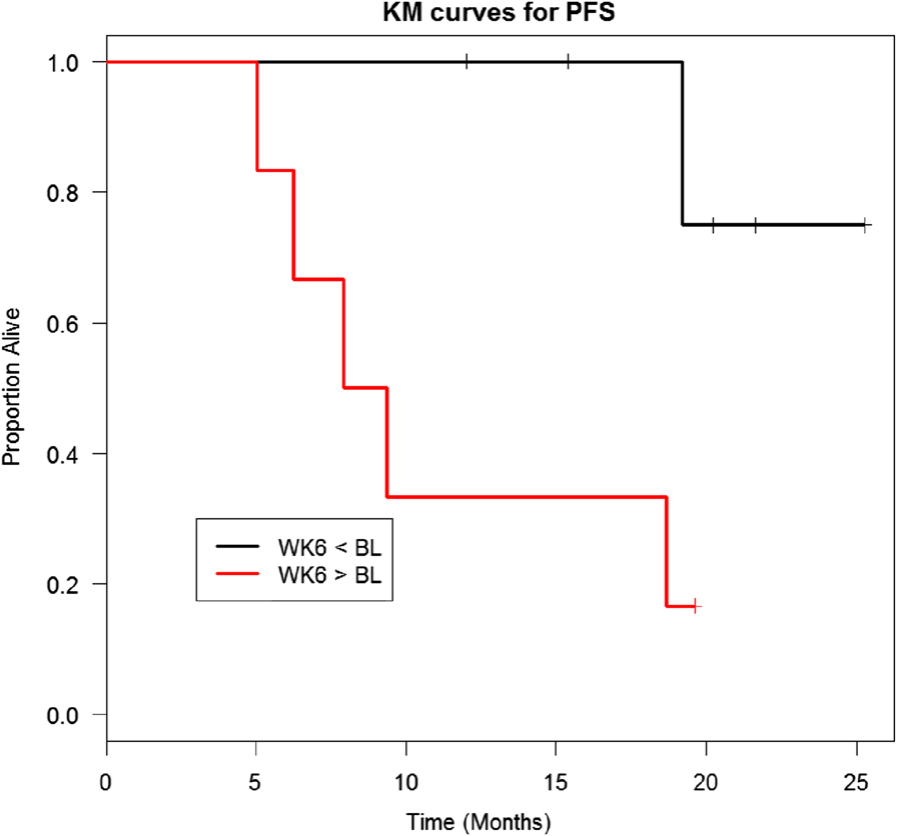
Association between reduced progression free survival with increase in circulating Treg. The patients were divided into two groups: those with % inhibition increased and those with % inhibition decreased. We defined the group by taking a majority of the three ratios. That is, if the results of at least 2 of the 3 ratios indicated a higher % inhibition at week 6 than at baseline, the patient was included in the WK6 > BL group, and if the results of at least 2 of the 3 ratios indicated a lower % inhibition at week 6 than at baseline, then the patient was included in the WK6 < BL group. Suppressive activity over time is shown in a Kaplan-Meier plot. Increase in Treg suppressive function was significantly associated with a decrease in PFS (*p* = 0.02381)

To understand the relationship between the suppressive function measured at the different Treg:Th ratios and the circulating frequencies, we investigated the association between them. We examined both CD4^+^CD25^hi^Foxp3^+^ and CD4^+^CD25^hi^CD39^+^ Treg cells. Foxp3 is an accepted marker of these cells; however, there is evidence that CD39 may be a more reliable marker of Treg [18]. CD4^+^CD25^hi^CD39^+^ T cells may have more immunosuppressive function through production of adenosine [19], and they have been found in increased levels in cancer patients [20]. A central finding of this study was that no statistically significant association between the change of Treg frequency after treatment and the Treg suppressive function (change of Treg proliferation inhibition post treatment) at any ratio was observed (Fig. 1). It was possible that increases or decreases in suppression after ipi might be mirrored in the circulating frequencies. However, there was also no statistically significant association between circulating Treg frequency and function tested at baseline and 6 weeks post-ipi (Fig. 2). The literature suggests that Treg mediated immune suppression has a negative impact on tumor control. Importantly, Treg suppressive function post-treatment (which was defined by taking a majority of three ratios, i.e. if the data from at least two of the three ratios showed Treg suppression post-treatment) was associated with a statistically significant decrease in PFS six weeks after treatment with ipi (*p* = 0.02381, Fig. 3). While an increase in Treg suppressive function over time was associated with a significant decrease in PFS, we did not see a correlation between the baseline proliferation inhibition and PFS.

## Discussion

We found that Treg suppression of autologous CD4+ T cell proliferation was changed after ipi treatment in a subset of patients, which did not mirror the circulating Treg phenotypic analysis performed. There were no significant overall trends across patients for ipi-induced Treg functional changes. The function of Treg in healthy individuals is to down-regulate the immune response and establish peripheral tolerance, guarding against autoimmunity. Treg suppress T cell function by several different mechanisms and to varying degrees, including suppression by secretion of inhibitory cytokines such as IL-10 and TGFβ; destruction of tumor cells via granzyme-A and perforin; metabolic disruption though adenosine nucleosides; and perhaps through targeting of dendritic cells [21]. It is thought that tumors manipulate the tumor microenvironment (TME) to induce Treg suppression of Th, and that different tumors may affect both the TME and Treg differently [22]. Questions have also been raised concerning whether Treg not only suppress the immune response under influence of tumor-derived factors, but also work to dampen inflammatory responses that would otherwise promote tumor growth, as increased Treg infiltration of tumor and in the peripheral blood has been associated with both poor and good prognosis, depending on the tumor [23]. A review of 124 studies of immune infiltration of the tumor itself demonstrated that Treg were considered to have a “good” effect on prognosis in a little over 30 % of studies and a “poor” effect in just over 40 % of studies; the remainder found no effect [24]. Part of the difficulty in determining the role of Treg in tumor immune response is the difficulty in identifying Treg *in vivo*. The precise phenotypic identification of Treg remains under discussion; however, there is recent consensus that identification can be made by establishing suppressive activity [25]. It is therefore possible that phenotypic measures of circulating Treg do not necessarily mirror functional changes. A limitation to this study is that there was not sufficient material from the tumor to compare the suppressive function of Treg in both peripheral blood and tumor. Likewise, we were not able to measure suppressive cytokines IL-10 and TGF-beta in the Treg suppression assay cultures.

Our finding that the post-ipi isolated Treg exhibited proliferation inhibition activity in vitro shows that they maintained their suppressive function. Treg suppressive activity did not significantly change before and after ipi at the highest tested ratios. This is in accord with previous studies, which have shown an increase in functional Treg in concert with a much greater increase in active Th following administration of an anti-CTLA-4 antibody, leading to the observed anti-tumor effect [26, 27]. In contrast, a separate study found that Th developed resistance to Treg inhibition after one month of treatment [28]. Another study similarly found that in vitro, at least, CTLA-4 blockade with tremelimumab negated the suppression of Th by Treg, more so at higher Th to Treg ratios. This may correlate with our finding of a significant decrease in Treg suppression of Th at 1:5 dilution. The study concluded that the effect of CTLA-4 was not due to an effect on Treg, but to activation of Th [29]. We previously also observed elevated numbers of Th and Treg following treatment with tremelimumab. We hypothesized that CTLA-4 blockade acts on Th to inhibit CTLA-4 suppression and allow greater expansion, leading to an antitumor response [30]. The association of an increase in circulating CD4^+^CD25^hi^Foxp3^+^ Treg at 6 weeks following CTLA-4 blockade with an increase in PFS may relate more to a concomitant and greater increase in the overall T cell population, of which Treg are a small fraction, than in the functionality of Treg. The impact of ipi on Th may outweigh its effect on Treg.

Therapeutic effects may depend on optimal CD8^+^ effector to Treg ratios [28]. We previously reported the detection of type I (interferon-γ producing), fully activated (CD69^+^) CD4^+^ and CD8^+^ antigen-specific T-cells (specific to gp-100, MART-1 and NY-ESO-1) that were significantly potentiated by ipilimumab [16]. Significant increases (3–10 fold) in CD3^+^/CD4^+^/INF-γ^+^ T-cells were observed only in patients who were progression free at 6-months. Moreover, Treg in tumor tended to be decreased post-ipi in responders. Therefore, what we see in the circulation may not be reflective of what happens in the TME. By testing Treg suppressive function, we observed trends of increase or decrease in suppressive function after ipi in individual patients that did not reach statistical significance over the entire group of patients. In this study, CD8^+^ cells were eliminated as part of the isolation process, and therefore we were unable to determine if they are less resistant to suppression than CD4^+^CD25^−^ cells. Another limitation to the study is that numbers are small.

Another limitation of the study is that we were unable to test CD4^+^CD25^+^Foxp3^+^ cells directly for functionality. A recent consensus statement by The Association for Cancer Immunotherapy (CIMT) immunoguiding program (CIP) concluded that minimally required markers of human Treg are CD3, CD4, CD25, CD127, and Foxp3 [31]. However, intracellular labeling of Foxp3 involves fixing the cells, thus making them unsuitable for further functional assays. Instead, we isolated a population of CD4^+^CD25^+^CD127^dim^ regulatory T-cells. CD127^dim^ is a marker for suppressor T cells. Using this method therefore may have selected for functional suppressor Treg rather than the entire population of CD4^+^CD25^+^Foxp3^+^ Treg. Another limitation is that in the phenotypic analysis, we did not surface stain for CTLA-4 expression.

## Conclusions

We found that Treg frequency measures do not correlate with suppressive activity measured ex vivo. However, Treg suppressive activity increases do correlate with poorer patient outcomes.

## Abbreviations

CFSE, carboxyl fluorescent succinimidyl ester; DFS, disease-free survival; FDA, Food and Drug Administration; IFN, interferon alpha-2b; Ipi, ipilimumab; MDSC, myeloid derived suppressor cells; OS, overall survival; PBMC, peripheral blood mononuclear cells; RFS, relapse-free survival; Teff, T effector cells; Th, T helper cells; TME, tumor microenvironment; Treg, T regulatory cells.

## Additional files

Additional file 1: Figure S1. Changes in Treg suppression of Th at 6 weeks compared to baseline. The bar graph on the left shows 1:1 Treg:Th ratio; the change was not significant (*p* = 0.1439). The middle bar graph shows 1:2 Treg:Th; the change was not significant (*p* = 0.782). The right bar graph shows 1:5 Treg:Th; the change was significant (*p* = 0.02557). (DOCX 3760 kb) Additional file 2: Table S1. Healthy donor control proliferation inhibition assays. (DOC 30 kb) Additional file 3: Table S2. Treg purification flow cytometry profile. (DOC 117 kb) Additional file 4: Figure S2. Representative flow cytometry plots. The top row shows flow cytometry results for Treg (CD25^+^CD127^dim/neg^) and Responders (CD4^+^CD25^-^CD127^dim/neg^). The middle row shows Negative Control and Positive Control flow cytometry plots. The bottom row shows an example suppression control for Treg:Responders at 1:1, 1:2, and 1:5 dilutions. (DOCX 342 kb)
