# Supplementary material for: Phenotypic and functional testing of circulating regulatory T cells in advanced melanoma patients treated with neoadjuvant ipilimumab
Source: J Immunother Cancer. 2016 Jun 21;4:38. doi: 10.1186/s40425-016-0141-1 (PMC4915044; doi:10.1186/s40425-016-0141-1)
Supplement: Additional file 1: Figure S1. — Changes in Treg suppression of Th at 6 weeks compared to baseline. The bar graph on the left shows 1:1 Treg:Th ratio; the change was not significant (p = 0.1439). The middle bar graph shows 1:2 Treg:Th; the change was not significant (p = 0.782). The right bar graph shows 1:5 Treg:Th; the change was significant (p = 0.02557). (DOCX 3760 kb) [file 40425_2016_141_MOESM1_ESM.docx]

Figure S1

Treg: CD4^+^**CD25^+^**CD127^dim/neg^

Responders: CD4^+^**CD25^-^**CD127^dim/neg^

Negative Control Positive Control

Suppresion control example, Treg:Responders

1:1

1:5

1:2
