# Supplementary material for: Phenotypic and functional testing of circulating regulatory T cells in advanced melanoma patients treated with neoadjuvant ipilimumab
Source: J Immunother Cancer. 2016 Jun 21;4:38. doi: 10.1186/s40425-016-0141-1 (PMC4915044; doi:10.1186/s40425-016-0141-1)
Supplement: Additional file 2: Table S1. — Healthy donor control proliferation inhibition assays. (DOC 30 kb) [file 40425_2016_141_MOESM2_ESM.doc]

Table S1. Healthy donor control proliferation inhibition assays.

| **Date** | **Condition** | **%CFSE-** |
| --- | --- | --- |
| Donor #1 | Positive | 93.0 |
|  | Negative | 2.0 |
|  | 1:1 | 31.0 |
|  | 1:2 | 68.7 |
|  | 1:5 | 86.5 |
| Donor #2 | Positive | 91.6 |
|  | Negative | 2.6 |
|  | 1:1 | 88.4 |
|  | 1:2 | 92.0 |
|  | 1:5 | 90.0 |
