# Supplementary material for: Phenotypic and functional testing of circulating regulatory T cells in advanced melanoma patients treated with neoadjuvant ipilimumab
Source: J Immunother Cancer. 2016 Jun 21;4:38. doi: 10.1186/s40425-016-0141-1 (PMC4915044; doi:10.1186/s40425-016-0141-1)
Supplement: Additional file 3: Table S2. — Treg purification flow cytometry profile. (DOC 117 kb) [file 40425_2016_141_MOESM3_ESM.doc]

Table S2. Treg purification flow cytometry profile.

| **Patient #** | **Time Point** | **Cell Type** | **%CD4^+^** | **%CD4^+^CD25^+^** | **Y MEAN total for CD25 MFI** |
| --- | --- | --- | --- | --- | --- |
| 1 | Baseline | Treg | 95.6 | 1.7 | 0.332 |
|  |  | Responders | 39.7 | 0.1 | 0.272 |
|  | Week 6 | Treg | 95.8 | 4.3 | 0.375 |
|  |  | Responders | 32.4 | 0.1 | 0.275 |
| 2 | Baseline | Treg | 94.8 | 0.8 | 0.318 |
|  |  | Responders | 63.2 | 0.2 | 0.312 |
|  | Week 6 | Treg | 97.9 | 2.6 | 0.346 |
|  |  | Responders | 83.1 | 0.3 | 0.315 |
| 3 | Baseline | Treg | 80.2 | 1.1 | 0.334 |
|  |  | Responders | 40.7 | 0.2 | 0.284 |
|  | Week 6 | Treg | 95.7 | 4.9 | 0.392 |
|  |  | Responders | 60.3 | 0.2 | 0.299 |
| 4 | Baseline | Treg | 91.7 | 3.3 | 0.385 |
|  |  | Responders | 42.4 | 0.3 | 0.288 |
|  | Week 6 | Treg | 92.6 | 4.1 | 0.387 |
|  |  | Responders | 63.1 | 0.4 | 0.298 |
| 5 | Baseline | Treg | 94.1 | 3.6 | 0.384 |
|  |  | Responders | 35.3 | 0.3 | 0.350 |
|  | Week 6 | Treg | 92.1 | 1.6 | 0.339 |
|  |  | Responders | 43.6 | 0.2 | 0.300 |
| 6 | Baseline | Treg | 93.0 | 3.7 | 0.337 |
|  |  | Responders | 41.5 | 0.2 | 0.352 |
|  | Week 6 | Treg | 92.8 | 1.9 | 0.330 |
|  |  | Responders | 56.2 | 0.2 | 0.346 |
| 7 | Baseline | Treg | 78.7 | 0.1 | 0.329 |
|  |  | Responders | 26.5 | 0.0 | 0.299 |
|  | Week 6 | Treg | 98.2 | 1.7 | 0.355 |
|  |  | Responders | 68.0 | 0.0 | 0.317 |
| 8 | Baseline | Treg | 98.6 | 0.3 | 0.305 |
|  |  | Responders | 76.9 | 0.3 | 0.325 |
|  | Week 6 | Treg | 90.4 | 0.4 | 0.323 |
|  |  | Responders | 72.2 | 0.1 | 0.312 |
| 9 | Baseline | Treg | 83.6 | 1.4 | 0.347 |
|  |  | Responders | 58.7 | 0.1 | 0.291 |
|  | Week 6 | Treg | 96.4 | 1.3 | 0.333 |
|  |  | Responders | 71.7 | 0.1 | 0.303 |
| 10 | Baseline | Treg | 94.5 | 2.5 | 0.359 |
|  |  | Responders | 21.6 | 0.2 | 0.338 |
|  | Week 6 | Treg | 67.5 | 3.7 | 0.342 |
|  |  | Responders | 38.6 | 0.3 | 0.281 |
| 11 | Baseline | Treg | 94.5 | 1.1 | 0.328 |
|  |  | Responders | 69.7 | 0.3 | 0.324 |
|  | Week 6 | Treg | 97.4 | 0.9 | 0.327 |
|  |  | Responders | 81.1 | 0.6 | 0.329 |
| 12 | Baseline | Treg | 97.5 | 0.3 | 0.331 |
|  |  | Responders | 16.5 | 0.1 | 0.286 |
|  | Week 6 | Treg | 94.5 | 1.4 | 0.335 |
|  |  | Responders | 54.6 | 0.5 | 0.326 |
| 13 | Baseline | Treg | 81.7 | 3.0 | 0.354 |
|  |  | Responders | 65.8 | 0.3 | 0.306 |
|  | Week 6 | Treg | 80.6 | 0.3 | 0.315 |
|  |  | Responders | 34.6 | 0.2 | 0.294 |
| 14 | Baseline | Treg | 95.7 | 3.1 | 0.357 |
|  |  | Responders | 8.9 | 0.0 | 0.260 |
|  | Week 6 | Treg | 90.7 | 7.4 | 0.442 |
|  |  | Responders | 45.9 | 0.2 | 0.341 |
| 15 | Baseline | Treg | 85.9 | 6.7 | 0.408 |
|  |  | Responders | 31.8 | 0.1 | 0.263 |
|  | Week 6 | Treg | 64.5 | 0.8 | 0.307 |
|  |  | Responders | 20.2 | 0.1 | 0.254 |
| 16 | Baseline | Treg | 95.4 | 2.5 | 0.357 |
|  |  | Responders | 65.5 | 0.3 | 0.312 |
|  | Week 6 | Treg | 98.4 | 1.9 | 0.342 |
|  |  | Responders | 85.6 | 0.4 | 0.307 |
| 17 | Baseline | Treg | 87.5 | 1.1 | 0.330 |
|  |  | Responders | 58.1 | 0.2 | 0.295 |
|  | Week 6 | Treg | 91.6 | 0.8 | 0.341 |
|  |  | Responders | 57.1 | 0.3 | 0.304 |
| 18 | Baseline | Treg | 90.9 | 0.6 | 0.326 |
|  |  | Responders | 31.5 | 0.1 | 0.274 |
|  | Week 6 | Treg | 90.4 | 0.9 | 0.325 |
|  |  | Responders | 67.4 | 0.4 | 0.311 |
