# Supplementary figures and images for: Phenotypic and functional testing of circulating regulatory T cells in advanced melanoma patients treated with neoadjuvant ipilimumab
Source: J Immunother Cancer. 2016 Jun 21;4:38. doi: 10.1186/s40425-016-0141-1 (PMC4915044; doi:10.1186/s40425-016-0141-1)

Figure S2


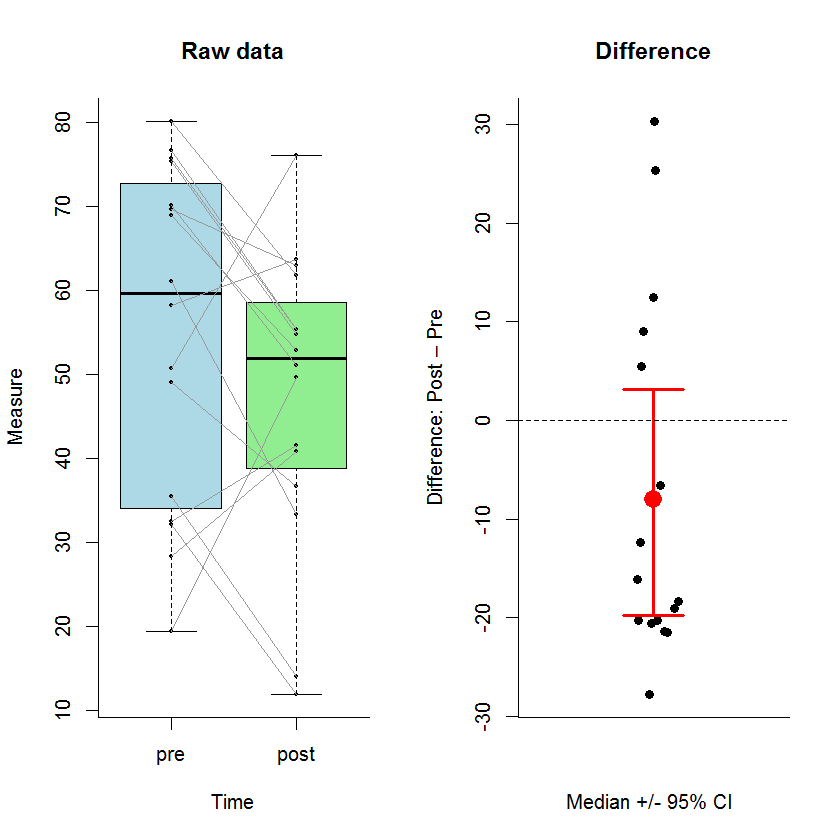

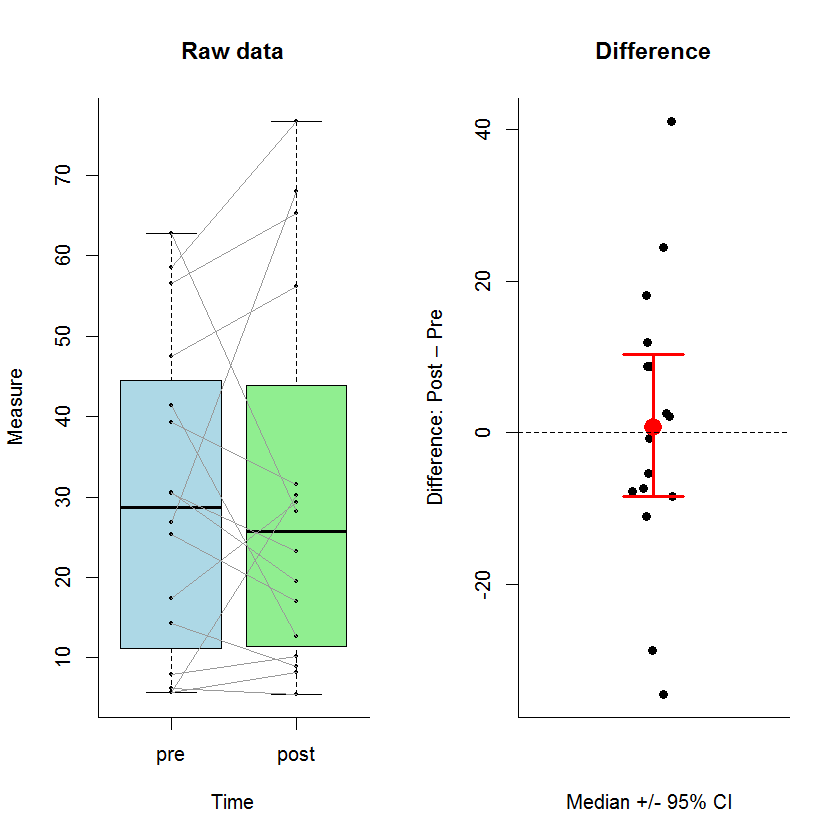

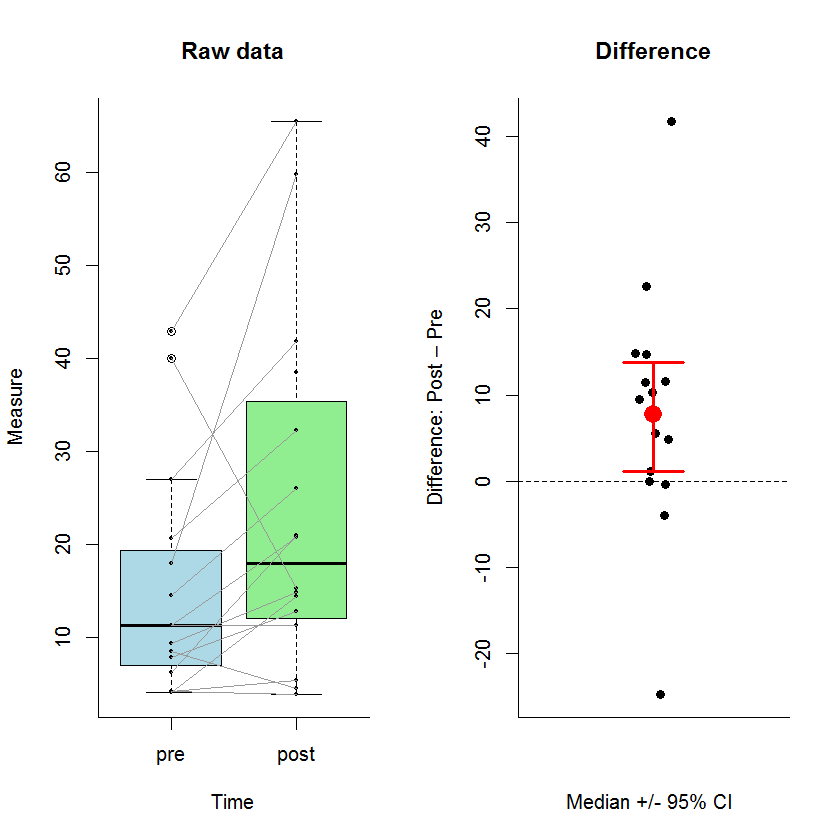

Supplement: Additional file 4: Figure S2. — Representative flow cytometry plots. The top row shows flow cytometry results for Treg (CD25+CD127dim/neg) and Responders (CD4+CD25-CD127dim/neg). The middle row shows Negative Control and Positive Control flow cytometry plots. The bottom row shows an example suppression control for Treg:Responders at 1:1, 1:2, and 1:5 dilutions. (DOCX 342 kb) [file 40425_2016_141_MOESM4_ESM.docx]
